# Plasma Retinol Kinetics and β-Carotene Bioefficacy Are Quantified by Model-Based Compartmental Analysis in Healthy Young Adults with Low Vitamin A Stores^1^,^2^

**DOI:** 10.3945/jn.116.233486

**Published:** 2016-08-10

**Authors:** Michael H Green, Jennifer Lynn Ford, Anthony Oxley, Joanne Balmer Green, Hyunjin Park, Philip Berry, Alan V Boddy, Georg Lietz

**Affiliations:** 3 Department of Nutritional Sciences, The Pennsylvania State University, University Park, PA; and; 4 Human Nutrition Research Centre and; 5 Northern Institute for Cancer Research, Newcastle University, Newcastle Upon Tyne, United Kingdom

**Keywords:** area under the curve, humans, bioefficacy, bioconversion, carotenoids, isotope dilution, retinoids, retinol kinetics, vitamin A deficiency, WinSAAM

## Abstract

**Background:** Model-based compartmental analysis of data on plasma retinol kinetics after administration of labeled retinol provides unique information about whole-body vitamin A metabolism. If labeled β-carotene is coadministered, its bioefficacy relative to the retinol reference dose can also be estimated.

**Objectives:** The objectives were to model plasma retinol kinetics after administration of labeled preformed vitamin A and provitamin A β-carotene and to determine relative β-carotene bioefficacy.

**Methods:** We used the Simulation, Analysis and Modeling software (WinSAAM version 3.0.8; http://www.WinSAAM.org) to analyze previously collected data on plasma [^13^C_10_]- and [^13^C_5_]retinol kinetics for 14 d after oral administration of 1 mg [^13^C_10_]retinyl acetate and 2 mg [^13^C_10_]β-carotene in oil to 30 healthy young adults of European ancestry [13 men, 17 women; mean ± SD age: 24.5 ± 4.2 y; mean ± SD body weight: 65.2 ± 10 kg; mean ± SD body mass index (in kg/m^2^): 22.5 ± 1.9] with moderate vitamin A intakes.

**Results:** A 6-component model provided the best fit to the data, including compartments for initial metabolism of vitamin A, plasma retinol, and extravascular vitamin A storage. The disposal rate was 6.7 ± 3.1 μmol/d, fractional catabolic rate was 6.0% ± 2.3%/d, and vitamin A stores were 123 ± 71 μmol. Relative β-carotene bioefficacy, based on the ratio of the areas under the fraction of dose curves calculated by WinSAAM, averaged 13.5% ± 6.02% (retinol activity equivalents = 7.7:1.0 μg). Interindividual variation in relative β-carotene bioefficacy was high (CV: 44%).

**Conclusions:** Vitamin A kinetics in these young adults were best described by essentially the same model that had been previously developed by using data for older adults with higher vitamin A stores; differences in parameter values reflected differences in vitamin A status. Estimated β-carotene bioefficacy was relatively low but similar to previously reported estimates obtained by graphical methods. This trial was registered at the UK Clinical Research Network as UKCRN 7413.

See corresponding commentary on page 1929 and article on page 2137.

## Introduction

Model-based compartmental analysis [specifically, the Simulation, Analysis and Modeling software (WinSAAM; http://www.WinSAAM.org) (1)] has been productively used to describe and quantitate retinol kinetics in rats over the past 35 y [see reference 2 for a review as well as Tan et al. (3)]. This approach has generated unique and important insights into whole-body vitamin A metabolism, including the fact that there is extensive recycling of retinol among plasma and tissues before irreversible utilization. On the basis of several small, retrospective analyses, general features of the vitamin A system are assumed to be the same in both rats and humans (2). In a published report based on a larger sample of humans, Cifelli et al. (4) presented a 6-component model for whole-body vitamin A metabolism in Chinese and American adults (*n* = 26). Results were based on plasma [^2^H_8_]retinol data collected for 56 d after the oral administration of deuterated retinyl acetate. Both groups in that study had moderate vitamin A stores, unlike the case in many lower-income populations worldwide for whom vitamin A deficiency and/or low vitamin A status are important public health problems.

In addition to information on vitamin A kinetics, WinSAAM can also be used to estimate relative β-carotene bioefficacy if a stable isotope of β-carotene is coadministered with labeled retinol. β-Carotene bioefficacy is defined as the proportion of ingested β-carotene that is absorbed and converted to retinol (5). The bioefficacy of a given β-carotene dose is estimated by dividing the area under the plasma isotope response curve (AUC)^8^ from time zero to infinity for labeled retinol derived from the β-carotene dose by the AUC for labeled retinol derived from the coadministered retinol reference dose. In previous studies (6–8), researchers used graphical methods to calculate AUCs from time zero to the end of their experiment and then calculated relative β-carotene bioefficacy as the ratio of the AUCs (“isotope reference method”). Note that such ratio methods reflect β-carotene absorption and bioconversion relative to the absorption and metabolism of the retinol reference dose. Because the literature indicates that β-carotene bioefficacy is affected by many factors (9), and varies widely depending on source, accurate methods for estimating β-carotene bioefficacy are needed.

In the current study, we applied model-based compartmental analysis to the data of Oxley et al. (10) on plasma retinol kinetics in a group of healthy young adults who had received single oral doses of [^13^C_10_]β-carotene and [^13^C_10_]retinyl acetate in oil. Our goals were to extend the knowledge on whole-body vitamin A metabolism in a population not previously studied and to compare model-generated estimates of β-carotene bioefficacy with graphical estimates. Because participants in the current study were selected to have moderate vitamin A intakes and were found to have relatively low vitamin A stores, our results may be generalizable to populations in lower-income countries who may be at risk of vitamin A deficiency.

## Methods

### 

#### Stable isotopes.

As reported by Oxley et al. (10), [^13^C_10_]retinyl acetate (8, 9, 10, 11, 12, 13, 14, 15, 19, 20-[^13^C_10_]retinyl acetate; molecular weight: 338.4) and [^13^C_10_]β-carotene (12, 12′, 13, 13′, 14, 14′, 15, 15′, 20, 20′-[^13^C_10_]β-carotene; molecular weight: 546.8) were purchased from Buchem BV. The isomeric composition of purchased [^13^C_10_]β-carotene was retrospectively determined to be 88.5% all*-trans*-β-carotene, 10.0% *cis*-β-carotene, and 1.5% α-carotene.

#### Subjects.

Healthy young adults of European ancestry were recruited by Oxley et al. (10). Subjects had not consumed β-carotene or multivitamin supplements containing vitamins A, C, and E during the 3 mo before the study and were excluded for the following reasons: being a smoker; being pregnant; having a BMI (in kg/m^2^) >30, high blood pressure, diabetes, lipid metabolic disorders, or liver, kidney, or gastrointestinal disease (as determined by a health and lifestyle questionnaire and blood test results); or consuming a diet high in preformed vitamin A (as determined by an FFQ). For the current modeling analysis, 15 of the 45 participants from the Oxley et al. study (10) were excluded because either their plasma isotope response curves did not follow the expected pattern of steady state tracer kinetics or there were anomalies in their data (see later in text). The study was conducted according to the guidelines set forth in the Declaration of Helsinki; written consent was obtained from each participant. All of the procedures were approved by the National Research Ethics Service, North East–Sunderland Committee (REC 09/H0904/20) before registration with the UK Clinical Research Network (UKCRN: 7413).

#### Study design.

Details on the 2-wk kinetic study were reported by Oxley et al. (10). Briefly, isotopes were separately dissolved by sonication in sunflower oil at concentrations of 2 g/L for [^13^C_10_]β-carotene and of 1 g/L for [^13^C_10_]retinyl acetate. One milliliter of each solution was administered to each subject (see below); thus, subjects received 2.954 μmol [^13^C_10_]retinyl acetate (“[^13^C_10_]retinol dose”) and 3.237 μmol [^13^C_10_]all-*trans*-β-carotene, 0.366 μmol [^13^C_10_]*cis*-β-carotene, and 0.055 μmol [^13^C_10_]α-carotene (“β-carotene dose”). Knowing that 1 molecule of all-*trans*-β-carotene could maximally be absorbed and converted into 2 molecules of retinol, whereas *cis*-β-carotene or α-carotene could yield only 1 retinol molecule, the value for the [^13^C_10_]β-carotene dose was calculated to be 6.895 μmol retinol activity equivalents (RAEs) (9).

For the kinetic study, volunteers consumed a standardized pasta meal, along with water, the evening before the experiment started and then fasted overnight. In the morning, a cannula was inserted in an antecubital vein in each subject. Subjects were sequentially given the doses of [^13^C_10_]β-carotene and [^13^C_10_]retinyl acetate orally via a syringe and then consumed a muffin and a yogurt smoothie (56% of total calories from fat). Blood was collected into 10-mL EDTA-coated evacuated tubes before administration of the dose (0 h) and at 2, 4, 6, 8, 10, and 12 h after dosing; cannulas were then removed. Subsequent fasting blood samples were obtained by venipuncture at 1, 2, 7, and 14 d. Blood samples were centrifuged at 1300 × *g* for 10 min at 4°C immediately after collection; plasma was stored at –80°C until analysis.

#### Analytical procedures.

Lipids including β-carotene and retinoids were extracted from plasma and analyzed by LC–tandem mass spectrometry (LC-MS/MS) according to the method of Oxley et al. (10). Briefly, 1 mL plasma was extracted by using 10 mL ethanol/ethyl acetate (50:50 vol:vol) with the use of [^13^C_10_]retinyl acetate and [^13^C_20_]β-carotene as internal standards. Solvent extracts were dried under a gentle stream of nitrogen, and the residues were reconstituted in 100 μL ethyl acetate. Analytes were separated by reverse-phase HPLC before MS/MS quantitation under atmospheric pressure chemical ionization in positive ion mode. Selected reaction monitoring of analytes was performed at *m/z* 547→330 and *m/z* 274→98 for [^13^C_10_]β-carotene and [^13^C_5_] cleavage products and at *m/z* 279→100 for metabolites of [^13^C_10_]retinyl acetate. [^12^C]Retinol in plasma was quantified by HPLC (11).

#### Kinetic analysis and parameters.

The fraction of the oral dose (FD) in plasma for both [^13^C_5_]retinol (derived from [^13^C_10_]β-carotene) and [^13^C_10_]retinol (derived from [^13^C_10_]retinyl acetate) at each time for each subject was calculated as follows:


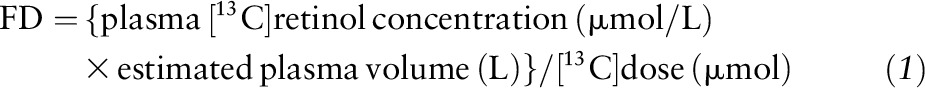


where estimated plasma volume (L) = body weight (kg) × 0.0435 (L/kg) (7). To accurately calculate [^13^C_10_]retinol and [^13^C_5_]retinol FD, background MS/MS analytical noise was subtracted for each sample.

**FIGURE 1:**
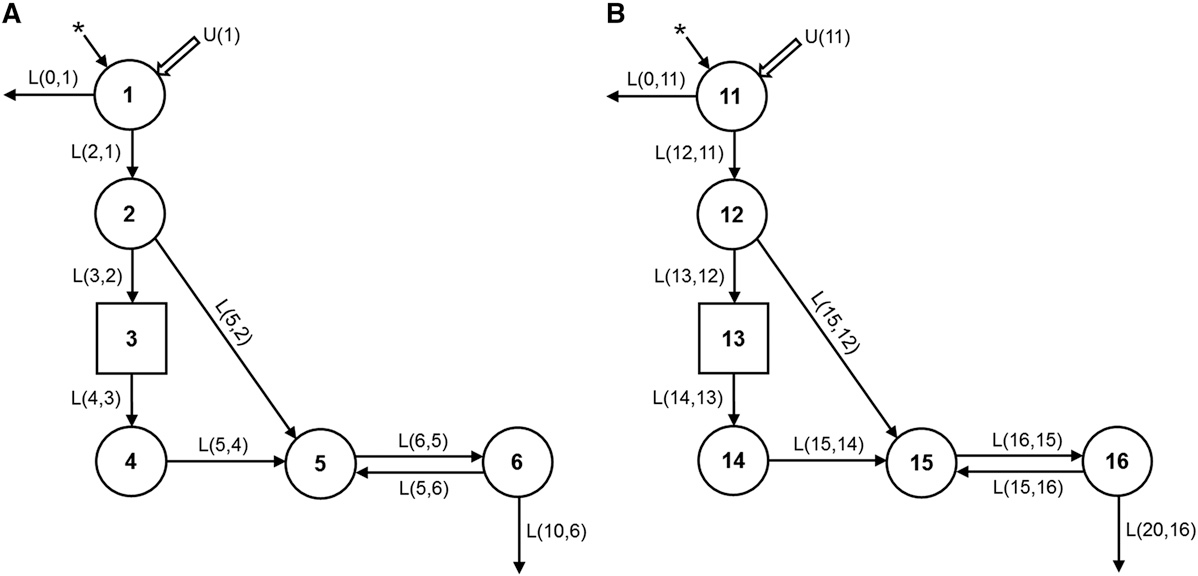
Proposed compartmental model for retinol kinetics in humans. Circles represent compartments; components 3 and 13, shown as rectangles, are delay elements, and interconnectivities between compartments [L(I,J)s] are fractional transfer coefficients or the fraction of retinol in compartment J that is transferred to compartment I each day. Panel A shows the model for [^13^C_10_]retinol and panel B shows the parallel model for [^13^C_5_]retinol (which was derived from the [^13^C_10_]β-carotene dose). In panel A, compartments 1–4 (including component 3) correspond to vitamin A digestion and absorption, loss of unabsorbed vitamin A, chylomicron production and metabolism, hepatocyte uptake of chylomicron remnant retinyl esters, and hepatic processing of retinol. Compartment 5 represents plasma retinol bound to retinol-binding protein and transthyretin; this retinol exchanges with vitamin A in 1 extravascular pool (compartment 6), which includes vitamin A stores in liver and other organs; thus, the fractional transfer of retinol to compartment 6 (stores) from compartment 5 (plasma) is L(6,5) (d^−1^) and the fractional transfer of retinol back to plasma from stores is L(5,6) (d^−1^). The asterisks (*) represent the site of input of the orally administered [^13^C] stable isotopes, and U(1) represents dietary vitamin A input. Absorption efficiency was fixed at 75%; thus, the fraction of the dose not absorbed is L(0,1) = 0.333 × L(2,1) because 25% is one-third of 75%. L(5,2) [and L(15,12) in the parallel model] represents the possibility that some retinol may be absorbed via the portal vein or picked up from the surface of chylomicrons; this parameter was required to fit the first data point. In panel B, compartments 11 and 12 represent β-carotene digestion and absorption; compartment 12 corresponds to the bioconversion of absorbed β-carotene to retinol in enterocytes. The remaining parts of the model are the same as in panel A, with L(16,15) = L(6,5), L(15,16) = L(5,6), and L(20,16) = L(10,6). L(I,J), fractional transfer coefficient. Adapted from reference 4 with permission.

By using WinSAAM (1), the data for plasma [^13^C_10_]retinol FD vs. time were fit to a model modified from the one developed by Cifelli et al. (4) to describe serum isotope data after the oral administration of [^2^H_8_]retinyl acetate to Chinese and American subjects. In this type of simple model, processes with similar kinetics are aggregated into the same compartment. In the current 6-component model (**Figure 1A**), compartment 1 is the site of introduction of the stable isotopes and dietary vitamin A into the system, as well as the site of loss of unabsorbed tracer. Retinol absorption efficiency was fixed at 75% on the basis of previous work in rats (12). Compartment 2 represents retinol in enterocytes; from there, retinol either transfers to delay component 3 or goes directly to plasma compartment 5. Delay component 3 is a WinSAAM device used here to accommodate the processes of chylomicron production and metabolism as well as the uptake of chylomicron remnants by hepatocytes. Compartment 4 corresponds to hepatic processing of retinyl esters from chylomicron remnants and hepatic secretion of retinol bound to retinol-binding protein (RBP). Compartment 5 represents retinol bound to RBP/transthyretin in plasma; this retinol exchanges with vitamin A in an extravascular pool (compartment 6). Compartment 6 comprises vitamin A stores and is the system’s site of irreversible loss of vitamin A [i.e., retinol that is converted to retinoic acid or other polar metabolites that cannot chemically convert back to retinol; represented here as L(10,6)]. In Figure 1A, the model parameters (fractional transfer coefficients [L(I,J)s]) correspond to the fraction of retinol in compartment J that is transferred to compartment I each day; for example, the fractional transfer of retinol to compartment 6 (stores) from compartment 5 (plasma) is L(6,5) (d^−1^) and the fractional transfer of retinol back to plasma from stores is L(5,6) (d^−1^). Parameters were systematically adjusted in WinSAAM to obtain a satisfactory fit of the data to the model for each subject. Final estimates for the L(I,J)s and their statistical uncertainties were generated by using weighted nonlinear regression analysis in WinSAAM. A fractional standard deviation (FSD) of 0.05 was used as the weighting factor.

The following kinetic parameters were then calculated by using the L(I,J)s for each individual [see Cifelli et al. (2) and Green et al. (13) for more details on these calculations]. The time for the retinol tracer to appear in plasma after the ingestion of the oral dose [mean time to plasma (time it takes for 50% of the absorbed tracer to reach plasma bound to RBP; TTP50%)] was calculated as the time at which 50% of the absorbed tracer was present in plasma compartment 5 (holoRBP). Plasma retinol pool sizes (μmol) were estimated from each individual’s mean plasma [^12^ C]retinol concentration during the study (determined by HPLC) and their estimated plasma volume. Then, plasma retinol pool sizes were used in a steady state solution in WinSAAM to estimate vitamin A pool sizes or traced mass [M(I); μmol]; disposal rate (μmol/d), or the rate of loss of vitamin A from compartment 6; and system fractional catabolic rate (FCR; d^−1^), or the fraction of retinol in compartment 6 that leaves irreversibly each day. Other parameters that were calculated include the half-life [t_1/2_(J)] for retinol in compartments 5 and 6, where t_1/2_(J) = ln 2/∑L(I,J)s exiting compartment J. The mean residence time [T(I,J)] is the mean time the tracer spends in compartment I after entering the system via compartment J; thus, residence time in plasma for absorbed retinol is T(5,5) and residence time in stores is T(6,5). T(SYS) [T(5,5) + T(6,5)] is the total time the tracer spends in plasma and stores from the time it enters plasma until it irreversibly leaves the system. The recycling number [v(5)] is the average number of times a retinol molecule recycles to plasma before irreversible loss; v(5) = {[T(5,5)/t_1/2_(5)] – 1}. Finally, the recycling time to plasma [tt(5)] was calculated as [T(6,5)/v(5)].

Once the data had been modeled for [^13^C_10_]retinol, a parallel model was formulated (see Results) for plasma [^13^C_5_]retinol (i.e., labeled retinol derived from the [^13^C_10_]β-carotene dose) and WinSAAM was used as above to calculate additional model parameters as well as relative β-carotene bioefficacy.

#### β-Carotene bioefficacy.

We used WinSAAM to calculate the relative efficiency of absorption and conversion of β-carotene to retinol. This was done by dividing the AUC for the FD in plasma β-carotene–derived retinol integrated from time zero to infinity by the AUC for the FD in plasma retinyl acetate–derived retinol integrated from time zero to infinity. In WinSAAM, AUC is calculated as the L-inverse matrix, which is equal to the residence time for retinol in plasma after it enters the system orally [T(15,11) and T(5,1), respectively]. We also calculated relative bioefficacy by using the graphical method described by Tang et al. (6), in which AUCs from time zero to the end of the experiment (14 d) were estimated for each isotope by using KaleidaGraph (version 4.1; Synergy Software).

#### Statistical methods.

Data are presented as means ± SDs. Graphs were made by using GraphPad Prism (version 6; GraphPad Software), and statistical analyses were performed by using JMP Pro (version 12.1; SAS Institute). *P* < 0.05 was considered significant. Student’s paired *t* test and linear least-squares regression analysis were used to compare β-carotene bioefficacy calculated by using WinSAAM compared with KaleidaGraph. Pearson’s correlation coefficient (*r*) and Spearman’s rank correlation coefficient (*R*_s_) were determined.

## Results

### 

#### Participant characteristics.

Subjects (*n* = 30) were healthy young adults of European ancestry (13 men and 17 women) with a mean ± SD age of 24.5 ± 4.2 y, mean ± SD body weight of 65.2 ± 10 kg, and mean ± SD BMI of 22.5 ± 1.9. The recruitment strategy selected volunteers with moderate intakes of preformed vitamin A, as determined by using the EPIC (European Prospective Investigation into Cancer and Nutrition) FFQ (14). As shown in **Table 1**, volunteers consumed 461 ± 501 μg retinol and 4.6 ± 4.4 mg β-carotene/d; carbohydrate, fat, and protein contributed 49.4%, 32.4%, and 15.3%, respectively, to total energy intake. During the 14-d study, mean plasma retinol concentrations measured by HPLC were normal for all subjects (mean ± SD 1.53 ± 0.27 μmol/L; range: 1.13–2.32 μmol/L).

**TABLE 1:** Baseline characteristics, dietary intake, and fasting plasma concentrations in healthy young adults of European ancestry^1^

	Values
Subject characteristics	
Age, y	24.5 ± 4.0
Weight, kg	65.2 ± 10.0
BMI, kg/m^2^	22.5 ± 1.9
Fat, %	20.9 ± 8.1
Dietary intake	
Energy, kcal/d	2480 ± 698
Carbohydrate, g/d	307 ± 99.6
Fat, g/d	89.3 ± 32.5
Protein, g/d	94.9 ± 32.7
β-Carotene, μg/d	4610 ± 4460
Retinol, μg/d	461 ± 501
Plasma	
β-Carotene, μmol/L	0.55 ± 0.33
Retinol, μmol/L	1.53 ± 0.27
TGs, mmol/L	0.91 ± 0.37
Cholesterol, mmol/L	4.91 ± 0.91

1 Values are means ± SDs, *n* = 13 men and *n* = 17 women.

Data for 15 other subjects studied by Oxley et al. (10) were excluded from this analysis on the basis of the following criteria: *1*) data were missing at critical time points (*n* = 1) or the FD was negative after background subtraction (*n* = 3) or *2*) plasma isotope response curves did not follow the expected pattern of steady state tracer kinetics as evidenced by a positive terminal slope for [^13^C_5_]- and [^13^C_10_]retinol curves (*n* = 3), the 2 curves converged (*n* = 3) or diverged (*n* = 1) over time, or there was an abnormally high peak value for [^13^C_10_]retinol (*n* = 4).

#### Kinetic data and compartmental model for [^13^C_10_]retinol.

**FIGURE 2:**
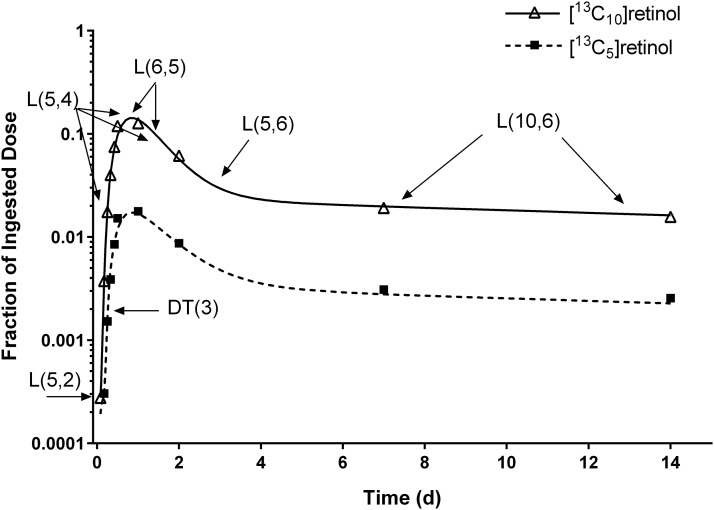
Model-predicted fractions of dose for [^13^C_10_]- and [^13^C_5_]retinol over time. Observed data (symbols) and model-predicted fraction of dose (lines) for [^13^C_10_]retinol (derived from the ingested [^13^C_10_]retinyl acetate dose) and for [^13^C_5_]retinol (derived from the ingested [^13^C_10_]β-carotene dose) compared with time in 1 representative healthy young-adult subject. Also indicated are times at which individual kinetic parameters are most sensitive to the data. The model is shown in Figure 1. DT(3), delay time in component 3.

Data for FD in plasma [^13^C_10_]retinol vs. time for 1 representative subject are plotted in **Figure 2** (upper curve); also shown is the model-simulated fit to the data. Plasma [^13^C_10_]retinol peaked at ∼12 h and then fell as tracer was distributed to extravascular tissues. The bend in the curve at ∼2 d indicates tracer recycling to plasma; the curve then enters a terminal slope, reflecting the system FCR for vitamin A. When fitting the data to the model shown in Figure 1, the model was sensitive to specific parameters at different times, as indicated in Figure 2. For example, L(5,4) and L(6,5) have their main impacts on the peak in the plasma retinol response profile and on the initial decrease after the peak, L(5,6) affects the bend in the curve from 2 to 6 d after dosing, and the terminal slope is most sensitive to L(10,6).

Mean model-predicted kinetic parameters for the 30 subjects are listed in **Table 2**. Three parameters related to the initial stages of the model [L(2,1), L(5,2), and L(15,12)] were not well identified as evidenced by large FSDs (data not shown); all others were well identified (mean FSD = 0.10 ± 0.042). The processes of chylomicron production and metabolism as well as the uptake of chylomicron remnants by hepatocytes took an average of 2.7 h (delay time in component 3 [DT(3)] × 24 h/d); 5.5 pools of plasma retinol were transferred to the extravascular compartment (compartment 6) each day; 17% of vitamin A in compartment 6 recycled to plasma each day, and 6.0% was irreversibly lost each day. By using the L(I,J)s for each individual along with their calculated plasma retinol pool size [mean M(5) = 4.3 ± 0.91 μmol], the following parameters were calculated by using a steady state solution in WinSAAM: M(6) = 123 ± 71 μmol, disposal rate (DR) = 6.7 ± 3.1 μmol/d, FCR(6,5) = 0.060 ± 0.023 d^−1^, FCR(5,5) = 1.6 ± 0.76 d^−1^, mean time to plasma (TTP50%) = 17 ± 3.0 h, t_1/2_(5) = 3.5 ± 1.4 h, t_1/2_(6) = 3.7 ± 1.6 d, T(5,5) = 0.79 ± 0.37 d, T(6,5) = 19 ± 7.4 d, T(SYS) = 20 ± 7.5 d, v(5) = 4.6 ± 2.1, and tt(5) = 4.8 ± 2.3 d.

**TABLE 2:** Model-predicted parameters for retinol kinetics in healthy young adults of European ancestry^1^

Parameter	Value
L(2,1)	24.2 ± 39.0
L(5,2)	0.114 ± 0.191
DT(3)	0.113 ± 0.0442
L(5,4)	2.98 ± 5.18
L(6,5)	5.50 ± 2.11
L(5,6)	0.169 ± 0.108
L(10,6)	0.0599 ± 0.0231
L(15,12)	0.110 ± 0.205
DT(13)	0.152 ± 0.0327
L(15,14)	1.43 ± 0.872
P(11)	10.9 ± 5.54

1 Values are means ± SDs, *n* = 30. Parameters are fractional transfer coefficients [L(I,J)] or the fraction of retinol in compartment J that is transferred to compartment I each day. For example, L(6,5) is the fractional transfer of retinol to compartment 6 (stores) from compartment 5 (plasma) (d^−1^) and L(5,6) is the fractional transfer of retinol back to plasma from stores (d^−1^). L(0,11), the fraction of the oral dose of β-carotene that was not absorbed, was calculated as P(11) × L(12,11), where P(11) is a constant. L(0,1) = 0.333 × L(2,1), L(16,15) = L(6, 5), L(15,16) = L(5,6), L(20,16) = L(10,6), and L(2,1) = L(3,2) = L(12,11) = L(13,12). The model is shown in Figure 1. DT(3) and DT(13), delay times in components 3 and 13, respectively.

#### Kinetic data and compartmental model for [^13^C_5_]retinol.

The lower curve in Figure 2 shows observed and simulated data for the FD in plasma [^13^C_5_]retinol (derived from the [^13^C_10_]β-carotene dose) vs. time in the same subject as plotted in the upper curve. Note that the 2 curves appear parallel from ∼12 h after dosing; this was true for all subjects in the current analysis.

By using the results from the [^13^C_10_]retinol data, we developed a parallel model for plasma [^13^C_5_]retinol (Figure 1B). The initial aspects of the 2 models (absorption and chylomicron metabolism) were independent, but the values for the parameters related to plasma retinol were the same in the [^13^C_5_]- and [^13^C_10_]retinol models; specifically, L(16,15) = L(6,5), L(15,16) = L(5,6), and L(20,16) = L(10,6). In the parallel model, compartment 11 represents β-carotene transit and absorption into enterocytes, and compartment 12 comprises the portion of absorbed β-carotene converted to retinol. Because a wide range of absorption and bioconversion efficiencies have been reported for β-carotene (15), we allowed irreversible loss of the unabsorbed dose [L(0,11)] to vary among individuals [L(0,11) = P(11) × L(12,11), where P(11) is a proportionality constant used to estimate the fraction of β-carotene not converted to retinol as determined from the [^13^C_5_]retinol data for each subject]. The vast majority of the β-carotene converted to retinol in enterocytes enters delay component 13, which represents the processes of chylomicron formation and metabolism and the uptake of chylomicron remnants by hepatocytes. The delay time in component 13 was 0.152 d × 24 h/d = 3.6 h (Table 2). As in the [^13^C_10_]retinol model, compartment 14 represents hepatocyte processing of chylomicron remnant retinol and hepatic secretion of retinol bound to RBP, compartment 15 comprises plasma retinol bound to RBP, and compartment 16 represents the vitamin A in extravascular stores that exchanges with retinol in compartment 15 and is the site of irreversible loss.

#### β-Carotene bioefficacy.

**FIGURE 3:**
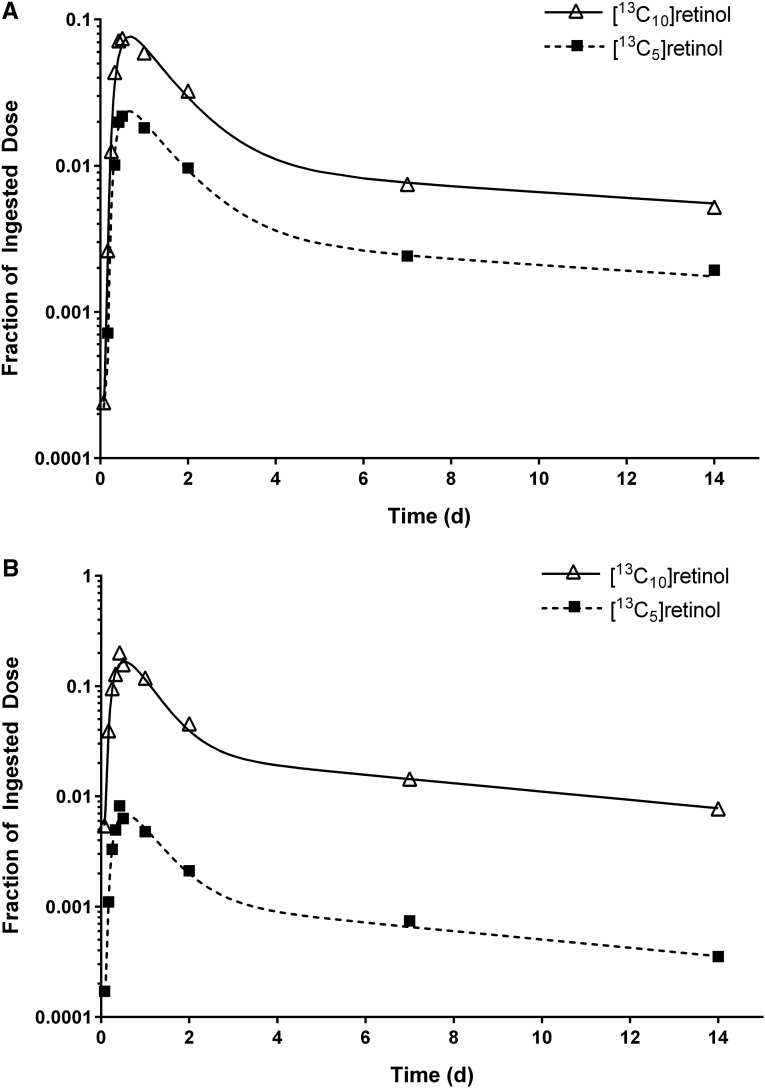
Fractions of dose in plasma [^13^C_10_]retinol (derived from ingested [^13^C_10_]retinyl acetate) and [^13^C_5_]retinol (derived from the ingested dose of [^13^C_10_]β-carotene) vs. time for the subject with the highest (A) and lowest (B) β-carotene bioconversion efficiency. Symbols correspond to observed data and lines correspond to model simulations; the model is shown in Figure 1.

**FIGURE 4:**
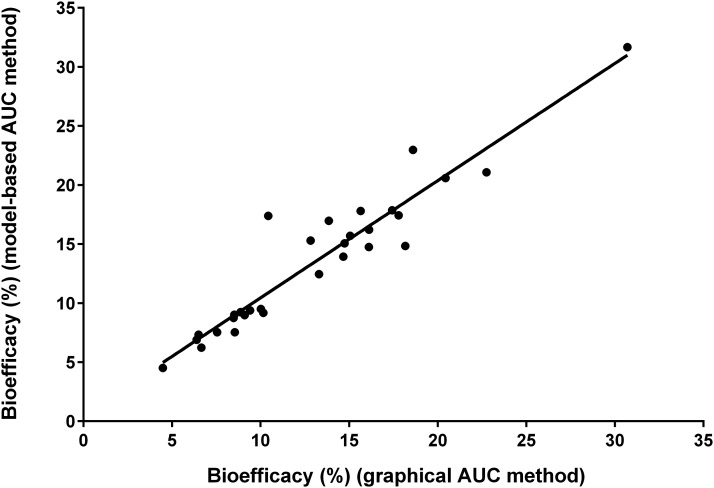
β-Carotene bioefficacy calculated in WinSAAM (model-based AUC method; http://www.WinSAAM.org) compared with KaleidaGraph (graphical AUC method; Synergy Software) for 30 subjects. Bioefficacy estimates were not significantly different (*P* = 0.23). The least-squares regression line (*y* = 0.531 + 0.992*x*) indicates a positive linear relation (*r* = 0.95, *P* < 0.001). AUC, area under the plasma isotope response curve.

Mean β-carotene bioefficacy calculated by using WinSAAM was 13.5% ± 6.02% (range: 4–32%) or 7.7:1.0 μg RAEs. There was extensive interindividual variation in β-carotene bioefficacy (CV: 44%). The subject whose data are plotted in Figure 2 had a bioefficacy of 14%; over all subjects, the closer the curves, the higher the bioefficacy (**Figure 3**). Values calculated by WinSAAM were not significantly different from those determined by using KaleidaGraph (13.1% ± 5.75%; range: 4.5–31%; *P =* 0.23). Least-squares linear regression analysis (**Figure 4**) indicates that estimates generated by the 2 methods were significantly correlated (*r* = 0.95, *R*_s_ = 0.93; *P* < 0.001).

## Discussion

In this report, we add to the existing knowledge on whole-body vitamin A metabolism in humans through the application of model-based compartmental analysis to the data of Oxley et al. (10), and we use modeling as an independent approach to compare with graphical methods for estimating β-carotene bioefficacy. To our knowledge, our analyses provide new information on retinol kinetics in a relatively large sample (*n* = 30) of healthy young adults (mean age: 24 ± 4 y) of European ancestry. We found that the structure of the 6-component model developed by Cifelli et al. (4) for older Chinese and American adults [54 ± 4 y (*n* = 14) and 59 ± 9 y (*n* = 12), respectively] provided a good fit to the current data, with a minor modification needed in the site of loss of the unabsorbed oral doses and with adjustment of the parameter values. Modeling revealed that total body stores of vitamin A were lower (123 ± 71 μmol) in the current Newcastle volunteers than in the Chinese (233 ± 109 μmol) or American (892 ± 637 μmol) subjects. Although the Newcastle subjects were selected for their low to moderate vitamin A intakes, our analysis further underscores their relatively low vitamin A status, in that they had only 18 d of vitamin A stores (compared with 42 d in the Chinese and 61 d in the Americans), where “days of stores” was calculated as model-predicted micromoles of total body stores/disposal rate (μmol/d). Note that, as was the case in the Chinese and American subjects, plasma retinol concentrations were normal in the Newcastle volunteers (1.54 ± 0.28 μmol/L). That is, there was a high prevalence of low total body stores of vitamin A in the Newcastle cohort despite normal plasma retinol concentrations. Similar findings of low vitamin A stores and normal plasma retinol concentrations have been reported in both adult Bangladeshi men [60 μmol (16)] and in Thai children [108 μmol (17)]. Our results show that this combination can also be found in a European group of young adults with low to moderate intakes of preformed vitamin A. Interestingly, although the current study was much shorter (14 d) than earlier investigations (4), which were carried out for 53–56 d, when we modeled the first 14 d of data for the American subjects in that previous study, the average model-predicted total body vitamin A stores were only ∼9% lower than those determined on the basis of data for the full experimental period, and values were identical for several individuals. This indicates that a shorter study, perhaps ∼28 d, may be adequate for estimating total body vitamin A stores on the basis of a compartmental model.

Comparing other model parameters between the 2 studies, retinol recycling time was much lower in the Newcastle subjects [tt(5) = 4.8 d] than that determined for individuals studied by Cifelli et al. (4) [tt(5) = 18–20 d], due to the former group’s lower vitamin A stores. In contrast, the number of times an average retinol molecule recycled from tissues to plasma before it was irreversibly utilized (recycling number) was similar in the 3 groups [v(5) = 4] but lower than that calculated for rats [v(I) = 8–11] (18, 19). The model-predicted disposal rate in the Newcastle subjects (6.7 ± 3.1 μmol/d) fell between that calculated for the Chinese (5.6 ± 2.0 μmol/d) and American (14.7 ± 5.9 μmol/d) subjects, and the system FCR was higher in the Newcastle group (6.0% ± 2.3%/d compared with 2.6% ± 0.8% and 2.0% ± 0.7%/d). These differences are likely attributable to the differences in stores and the duration of the current study.

It is worth mentioning that, in the current subjects, the kinetics of [^13^C_10_]retinol (from the [^13^C_10_]retinyl acetate dose) and [^13^C_5_]retinol (from the [^13^C_10_]β-carotene dose) were the same once retinol entered plasma bound to RBP. That is, based on visual inspection, the plasma response curves were parallel from ∼12 h after dosing (Figure 2). The fact that the curves are parallel indicates both that retinol from the 2 sources was handled equivalently after secretion from the liver on RBP and that there were no isotope effects due to the different number of [^13^C] atoms (10 compared with 5) in retinol from the 2 stable isotope doses.

The relative disposition of the plasma isotope response curves also led us to hypothesize that there might be a more direct way to estimate β-carotene bioefficacy than from the ratio of the AUCs. Specifically, the ratio of the 2 stable isotopes in a given plasma sample may provide a reliable index of bioefficacy, as suggested earlier by Hickenbottom et al. (20). In the current subjects, the mean plasma retinol isotope ratio on day 2 was 12.5% ± 5.74% (compared with 13.5% on the basis of WinSAAM). Further work will be needed to explore the validity of a plasma isotope ratio method for estimating β-carotene bioefficacy; however, if it is confirmed, it would allow the estimation of this factor on the basis of analysis of a single blood sample rather than requiring a relatively long-term study with multiple blood samples (which is needed to estimate AUC).

Although β-carotene bioefficacy varied widely among individuals in this study (CV: 44%), we found that estimates generated by graphical analysis as described by Tang et al. (6) were confirmed by WinSAAM. Mean bioefficacy from a physiologic dose of β-carotene (2 mg) in oil was 13.5% in the current subjects. This estimate is similar to values reported by Tang (21) for subjects who were given 1.5 mg [^2^H_8_]β-carotene in oil (14%; RAEs: 7.4:1.0 μg) and those determined for the American and Chinese subjects studied by Tang and colleagues (6, 7) (11% bioefficacy; RAEs: 9.1:1.0 μg) after doses of 6 mg deuterated β-carotene in oil. However, relative β-carotene bioefficacy calculated for the current subjects is lower than that predicted by the FAO/WHO (30%; RAEs: 3.3:1.0 μg) and by the US National Academy of Medicine (50%; RAEs: 2.0:1.0 μg) for β-carotene in oil (22, 23). Because β-carotene bioefficacy is affected by many experimental factors, including size of dose, dosing regimen, and food matrix (9), as well as by interindividual subject factors such as vitamin A status and genetic variations in the β-carotene 15,15'-dioxygenase 1 (BCO1) cleavage enzyme (6, 24, 25), much more work is needed to understand differences in absorption and metabolism of β-carotene and other carotenoids.

In conclusion, the application of model-based compartmental analysis has the potential to provide unique information and insights into vitamin A and β-carotene kinetics and metabolism as well as practical results with the potential for application in the field. For example, in the accompanying article in this issue (26), we used modeling results to modify the original retinol isotope dilution equation (27) for predicting total body vitamin A stores on the basis of a single, early blood sample. Similarly, our current modeling results confirm graphical methods for estimating β-carotene bioefficacy and may lead to a simpler method that will be useful in the field.

## Footnotes

8 Abbreviations used: AUC, area under the plasma isotope response curve; FCR,fractional catabolic rate; FD, fraction of the oral dose; FSD, fractional standarddeviation; L(I,J), fractional transfer coefficient; MS/MS, tandem mass spectrometry;RAE, retinol activity equivalent; RBP, retinol-binding protein.
